# Malignant Tumours Presenting as Chronic Leg or Foot Ulcers

**DOI:** 10.3390/jcm10112251

**Published:** 2021-05-22

**Authors:** Frédéric Toussaint, Michael Erdmann, Carola Berking, Cornelia Erfurt-Berge

**Affiliations:** 1 Department of Dermatology, Universitätsklinikum Erlangen, Friedrich-Alexander-University Erlangen-Nuremberg (FAU), Ulmenweg 18, 91054 Erlangen, Germany; frederic.toussaint@uk-erlangen.de (F.T.); michael.erdmann@uk-erlangen.de (M.E.); carola.berking@uk-erlangen.de (C.B.); 2 Comprehensive Cancer Center Erlangen-European Metropolitan Area of Nürnberg (CCC ER-EMN), 91054 Erlangen, Germany

**Keywords:** basal cell carcinoma, chronic ulcer, melanoma, squamous cell carcinoma, venous leg ulcers

## Abstract

Our purpose was to collect data on the incidence of malignant skin tumours presenting as chronic leg or foot ulcers in a tertiary centre, and to analyse the frequency and type of initial clinical misdiagnoses in these cases. A retrospective chart review of cases with melanoma or other malignant neoplasms of the skin of the lower extremity treated in a tertiary centre during January 2010 until February 2020 was conducted to identify cases that presented as chronic ulcers. Out of 673 cases, 26 (3.9%) were identified with a total of 27 malignant tumours presenting as chronic ulcers of the lower leg or foot. Therefrom, seven were diagnosed as melanoma, eight as squamous cell carcinoma, and twelve as basal cell carcinoma. The mean interval until diagnosis for all tumour types was 44.4 months (median 24 months). A delay in correct treatment occurred in 12 out of 26 cases (46%) as a result of misdiagnosis with subsequent treatment as chronic leg or foot ulcers of a different etiology. Misdiagnoses were venous ulcer, traumatic wound, mixed arterial and venous ulcer, arterial ulcer, and ulcer of an unknown origin. Malignant ulcers presenting as chronic ulcers are rare, but often lead to misdiagnosis.

## 1. Introduction

Chronic leg ulcers are a common medical condition and may be caused by numerous factors. There is no precise data on the prevalence of chronic leg ulceration. According to the data of a German statutory health insurance in 2012, the prevalence of leg ulcers was 0.7% [1]. Most chronic leg ulcers have a vascular etiology. Venous insufficiency is the most common cause, followed by combined arterial and venous insufficiency and arterial insufficiency alone. Other causes are vasculitis, pyoderma gangrenosum, infectious diseases, calciphylaxis, drug reactions, and neoplasms [2]. Malignancy in chronic ulcers has to be differentiated in primary malignant ulcers and secondary malignancy because of the transformation of a chronic wound into a malignant tumour. Malignant transformation of chronic leg ulcers is a rare but possible condition, with squamous cell carcinomas (SCC) being the most common tumour type developing in chronic leg ulcers [3]. The prevalence of malignancy in chronic leg ulcers in the literature ranges from 2.0% to 2.8% in retrospective analyses [4,5,6] and 4% to 10.4% in prospective studies [7,8]. The two most common types of ulcerating skin tumours are basal cell carcinoma (BCC) and SCC [9]. There are case reports of other rarer skin cancer types imitating chronic leg ulcers, like cutaneous lymphoma [10], Kaposi’s sarcoma [11], eccrine porocarcinoma [12], and angiosarcoma [13]. Because of its rarity, malignant tumours as a cause of chronic leg or foot ulcers are often overlooked [14]. Initial misdiagnoses could lead to a delay in appropriate therapy management. There are no consistent clinical practice guidelines about the diagnostics of non-healing ulcers. A German S3 guideline for the local treatment of chronic wounds from 2013 recommends a biopsy in cases of atypical wounds and differential-diagnostic evaluation in cases of absence of healing tendency after six weeks of treatment, according to the guidelines [15]. The aim of our study was to collect data on the incidence of malignant skin tumours presenting as chronic leg or foot ulcers in a tertiary centre, and to analyse the frequency and type of initial clinical misdiagnoses in these cases.

## 2. Methods

### 2.1. Cases

**Figure 1:**
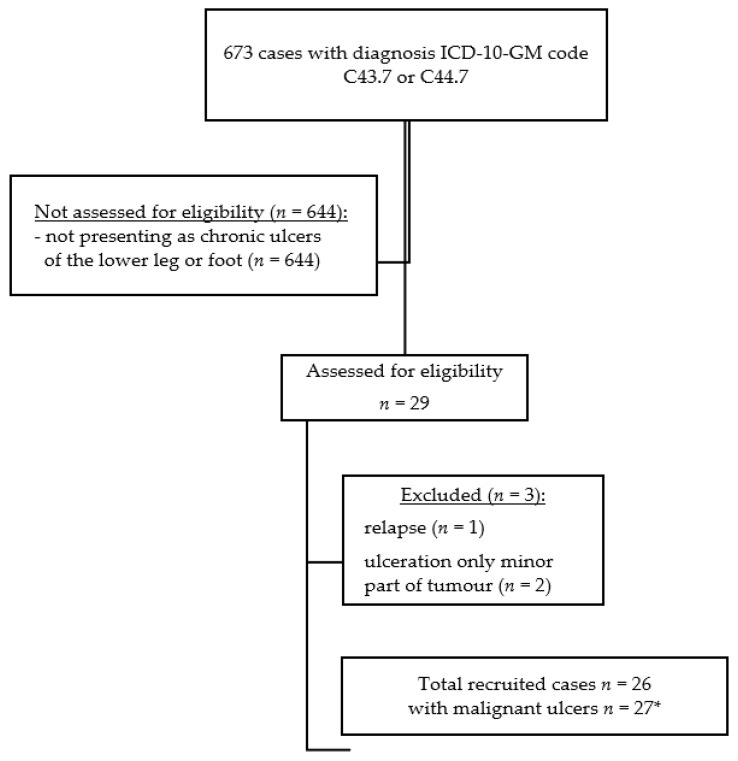
Flow chart of the study population. ICD-10-GM code (German version of the 10th version of the International Classification of Diseases) C43.7—melanoma of the lower limb. ICD-10-GM code C44.7—other malignant neoplasms of the skin of the lower limb. * one patient with two basal cell carcinomas presenting as chronic ulcers.

Cases with melanoma or other malignant neoplasms of the skin of the lower limbs treated in the Department of Dermatology, University Hospital Erlangen, Germany, during January 2010 until February 2020, were identified by the ICD 10 GM codes (German version of the 10th version of the International Classification of Diseases) C43.7 and C44.7 in the electronic patient database (Soarian^®^, Cerner Health Services, Berlin, Germany; Figure 1). Clinical findings at the day of initial presentation at our department and the medical history prior to this time point were reviewed from the patients’ data charts. Predefined inclusion criteria for further analysis were as follows: (i) clinical presentation as ulcer of the lower leg or foot persisting longer than 8 weeks (defining a chronic leg ulcer), (ii) histological confirmation, and (iii) photodocumentation. Exclusion criteria were tumour relapse, ulceration of only a minor part of the clinically evident tumour, and documented refusal of the patient to use his/her data. Final diagnosis was histologically confirmed either in our Department of Dermatopathology or by an external pathologist. Included patients were analysed for (a) patient characteristics (age and sex), (b) tumour characteristics (tumour type and location) and tumour stage at initial diagnosis, (c) time until correct diagnosis (interval between manifestation of ulceration recognized by the patient, relatives or health care providers and the date of correct diagnosis), (d) misdiagnosis prior to correct diagnosis and previous treatments, and (e) potential misleading comorbidities.

This retrospective data assessment includes data collected solely during regular patient care. Approval by the Ethics Committee was therefore not necessary.

### 2.2. Statistical Evaluation

Parameters were analysed with Microsoft^®^ Excel 2019 for descriptive statistical analysis.

## 3. Results

### 3.1. Case Characteristics

**Figure 2:**
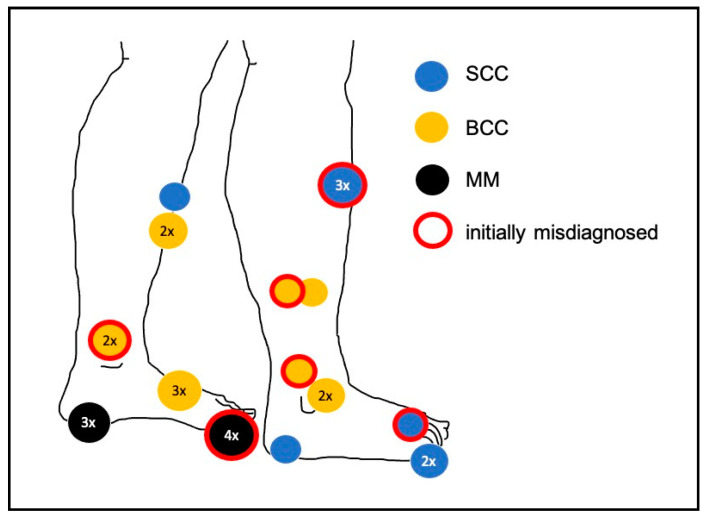
Locations of the malignant tumours presenting as chronic ulcers. For example, four misdiagnosed melanomas were situated on the hallux. SCC—squamous cell carcinoma; BCC—basal cell carcinoma; MM—melanoma.

**Figure 3:**
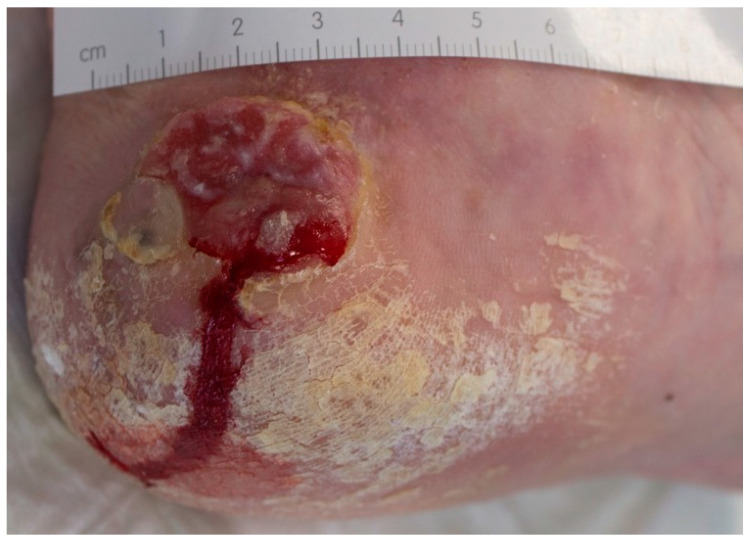
Example of a MM on the heel, initially misdiagnosed and treated as traumatic wound.

**Figure 4:**
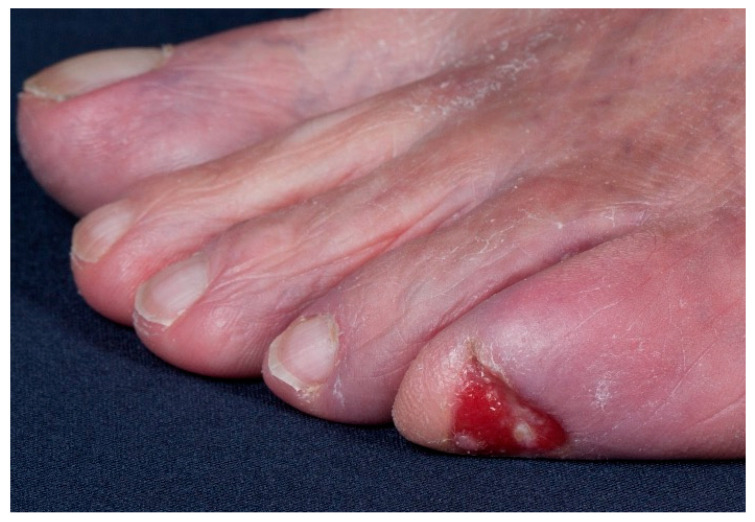
Example of an SCC on the toe, presenting as a chronic ulcer of an unknown etiology.

**Figure 5:**
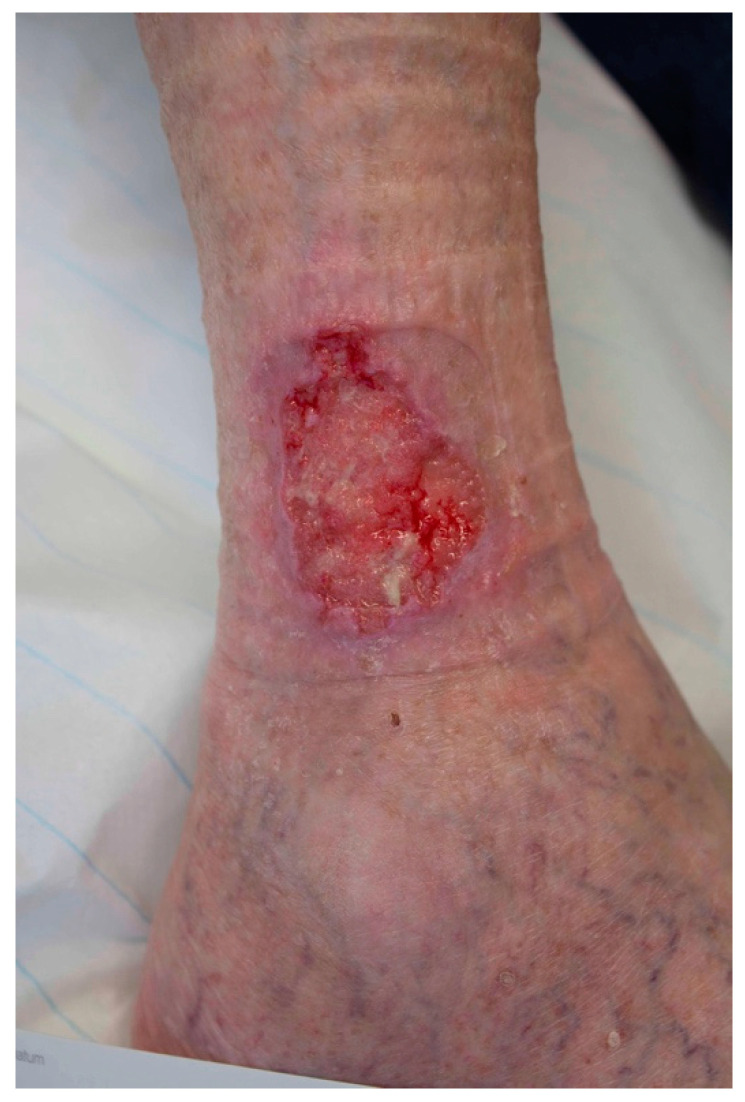
Example of a BCC on the lateral gaiter area, misdiagnosed as a chronic venous ulcer.

Out of the 673 cases with melanoma or other malignant neoplasms of the skin of the lower limb, 26 cases (3.9%) were identified with a total of 27 malignant tumours presenting as chronic ulcers of the lower leg or foot (Figure 2). Twelve cases were female (46%) and fourteen (54%) were male. The mean age at first diagnosis was 78 years (median 80 years), with a range of 57 to 97 years (Table 1). Out of the 27 malignant ulcers, seven were diagnosed as melanoma (26%; Figure 3), eight as squamous cell carcinoma (SCC; 30%; Figure 4), and twelve as basal cell carcinoma (BCC; 44%; Figure 5). All melanoma cases presented as amelanotic melanoma. For 5 out of 26 cases, pain was documented as a symptom of the ulceration.

### 3.2. Tumour Stage at Diagnosis

The mean diameter of 27 chronic ulcers upon diagnosis as malignant tumours was 4.1 cm (median 3 cm), with a range of 0.4 to 15 cm. The seven chronic ulcers finally diagnosed as melanoma showed a mean diameter of 2.5 cm (median 2.5 cm), with a range of 1.5 to 5 cm. The mean tumour thickness of the melanomas at initial diagnosis was 5.5 mm (median 4.8 mm), with a range of 2.5 to 12 mm. Thus, local tumour disease at initial diagnosis according to the American Joint Committee on Cancer (AJCC) 2017 classification for melanoma was at least stage IIB (T3b and T4b). Four of these seven melanoma cases (57%) already presented with advanced disease, including satellite, in-transit, or lymph node metastases, resulting in melanoma stage IIIC (*n* = 3) and IIID (*n* = 1) AJCC 2017. None of the melanoma cases suffered from distant metastases at the initial diagnosis. Eight chronic ulcers were proven to be SCC, with a mean diameter of 3.9 cm (median 3.5 cm) with a range of 1 to 8 cm. The mean tumour thickness of SCC at initial diagnosis was 2.5 mm (median 2.3 mm), with a range of 1 to 4.8 mm. Six out of eight (75%) SCC were high-risk tumours because of one or more of the following parameters: diameter > 2 cm (*n* = 4), poorly differentiated tumours (*n* = 3), and immunosuppression with cyclosporine (*n* = 1). The twelve chronic ulcers finally diagnosed as BCC showed a mean diameter of 5.1 cm (median 3.3 cm), with a range of 0.4 to 15 cm. The mean tumour thickness of BCC at initial diagnosis was 3.3 mm (median 3.1 mm), with a range of 1.5 to 6 mm. Nine out of twelve (75%) BCC were sclerosing BCC.

### 3.3. Misdiagnosis and Misleading Factors

In 14 out of 26 cases (54%), no earlier medical consultation was reported until the initial, histologically confirmed tumour diagnosis. Delay in correct treatment occurred in 12 out of 26 cases (46%) due to misdiagnosis with subsequent treatment as chronic leg or foot ulcers of a different etiology (Table 2). Patients reported a trauma leading to a chronic wound in 10 out of 27 cases (37%), of which 4 out of 7 were melanoma cases (57%), 4 out of 12 were BCC cases (33%), and 2 out of 8 were SCC cases (25%). Further potentially misleading comorbidities were chronic venous insufficiency (*n* = 7), peripheral arterial occlusive disease (*n* = 6), radioderma (*n* = 1), erosive lichen planus (*n* = 1), and polycythaemia vera (*n* = 1). Four cases of BCC and one case of SCC were previously diagnosed as chronic venous leg ulcers because of a known chronic venous insufficiency, and were treated accordingly with compression therapy and professional wound care management. In one of these cases, the patient received stripping of the great saphenous vein and meshgraft transplantation two years before the diagnosis of BCC. Because of a non-healing wound despite meshgraft transplantation, a new meshgraft transplantation was planned. A histopathological examination of the ulcer ground prior to the new transplantation revealed BCC. Despite two external biopsies from the border area ruling out the malignancy of a chronic venous leg ulcer persisting for 24 months, an additional biopsy 60 months later detected SCC. One case with both chronic venous insufficiency and peripheral arterial occlusive disease was treated with iliac and femoral artery thrombectomy, angioplasty, and stripping of the great saphenous vein 98 months prior to the diagnosis of SCC. One case with melanoma of the hallux was treated as a chronic arterial ulcer and received an angioplasty of the femoral artery two months prior to the diagnosis of melanoma. Two cases of melanoma and one case of SCC were treated as chronic wounds due to trauma with professional wound care management prior to the tumour diagnosis. One case of melanoma and one case of SCC were treated as chronic ulcers of an unknown etiology with professional wound care management prior to the final diagnosis. In cases of initial misdiagnoses, patients were seen by either general practitioners; general surgeons; vascular surgeons; professional wound care managers; or, in one case, by a dermatologist, prior to correct diagnosis. Prior biopsies were only conducted in one case, as described above.

### 3.4. Delay in Diagnosis

The mean interval between the manifestation of ulceration recognized by the patient, relatives, or health care providers as well as the date of initial diagnosis for all tumour types was 44.4 months (median 24 months), with a range of 4 to 240 months. In the cohort of initially misdiagnosed cases, the mean interval until correct diagnosis was 63.4 months (median 24 months), with a range of 5 to 240 months. In the cohort of initial correct diagnosis, the mean interval to diagnosis was 28.1 months (median 18 months), with a range of 4 to 120 months. The mean interval from the onset of symptoms until melanoma diagnosis was 14.6 months (median 12 months), with a range of 6 to 24 months. For the subgroup of SCC, the mean interval until initial diagnosis was 65 months (median 33 months), with a range of 3 to 240 months. For the subgroup of BCC, the mean interval until initial diagnosis was 48.5 months (median 24 months), with a range of 5 to 120 months.

## 4. Discussion

A considerable proportion of all malignant skin lesions of the lower leg imitates a chronic leg or foot ulcer: 3.9% of our case cohort presented with chronic leg or foot ulcers and were finally diagnosed as malignant tumours of the skin. This mimicry led in 46% of the patients to an initial misdiagnosis and a subsequent delay in treatment.

In the majority of previous studies, BCC was the most common type of malignancy found in chronic leg ulcers, followed by SCC [4,5,6,7,8]. This is in accordance with our findings. Of all three tumour types presenting as chronic ulcers in the lower legs, BCC was the cause in 44% and SCC in 30%, reflecting the general incidence of malignant skin cancer [16]. In our observation period, we found no other rare non-melanoma skin cancers that are also included in the ICD-10-GM code C44.7, like porocarcinoma or Kaposi’s sarcoma, presenting as chronic ulcers.

Malignancy in chronic ulcers has to be differentiated in primary malignant ulcers and secondary malignancy. Baldursson B et al. estimated a rate of 0.21% of malignant transformation of chronic venous leg ulcers to SCC, with a mean duration of the ulcer prior to SCC diagnosis of 24.5 years [17]. Only few cases of BCC or melanoma arising in chronic leg ulcers have been reported [18,19]. However, some of these cases have to be interpreted with caution, namely that clinical misdiagnoses led to the assumption of a secondary malignancy. Therefore, in the literature, most authors call for a minimum interval of at least 3 years between the diagnosis of a chronic ulcer and the transformation into a SCC, and they call for a previous negative biopsy in cases of transformation into a BCC [17,19,20]. In our patient collective, we observed two cases of transformation to SCC. One of these ulcers had a duration of 60 months and a negative biopsy 36 months prior to the diagnosis of SCC. The other ulcer already existed for 240 months prior to diagnosis.

Our data confirm a great diagnostic delay in cases of tumours presenting as chronic leg ulcers. In the cohort of initial correct diagnosis, the mean interval to diagnosis was 28.1 months (median 18 months) and in the cohort of initially misdiagnosed cases it was 63.4 months (median 24 months). Even when excluding the two cases of potential secondary malignancy, the mean delay in diagnosis was still 46.1 months (median 21 months). A significant delay is caused by the late decision of the patient to seek medical advice. One reason may be the mean age of 78 years for our patients who required the aid of relatives or nurses. Secondly, individual attempts to explain the symptoms may have led to a delay in consulting a physician. In our study, in 37% of cases, the chronic ulcers that proved to be a malignant tumour were trivialized to a preceding trauma by the patients.

The mean elapsed time until melanoma diagnosis in our study was 14.6 months (median 12 months). Sondermann et al. showed a slightly shorter median delay of 9 months for the initial diagnosis of melanoma located on the foot, but they included all subtypes of melanoma. Ulcerated melanomas led more frequently to misdiagnosis and a significant increased delay in diagnosis of 365 days compared with 92 days without ulceration [21]. In our study, 57% of all melanomas were initially misdiagnosed as chronic wounds due to trauma, peripheral arterial occlusive disease, or an unknown aetiology. The hallux and the heel were the primary sites of the melanoma cases and were thus more easily exposed to potential injuries or abuse of footwear [22]. Other groups found a rate of misdiagnosis of melanoma located on the foot between 25 and 34% [21,23,24,25], not only considering melanomas presenting as chronic ulcers. Sondermann et al. [21] showed a significantly reduced calculated five-year overall survival rate of 63.5% in the cohort of misdiagnosed melanoma compared with 88.4% in the cohort of initial correct diagnosis. The mean tumour thickness of our melanoma cases presenting as chronic ulcers was 5.5 mm (median 4.8 mm), which was higher than previously reported by Sondermann et al. [21] (mean tumour thickness of 2.6 mm and median of 1.6 mm, *n* = 107), Fortin et al. [23] (mean 3.0 mm, *n* = 60), and Soon et al. [25] (mean 3.3 mm, *n* = 53). The majority of our melanoma cases (57%) developed advanced disease according to AJCC 2017 [26] stage IIIC and IIID at diagnosis, which was more common compared with the cohort of Sondermann et al. [21] with 23.1% in stages III–IV. Thus, melanomas located on the foot and presenting as chronic wounds are prognostically worse than other types of melanoma of the foot. This is not unexpected, as the melanomas in our cohort were all amelanotic and ulcerated.

The mean delay until BCC diagnosis in our study was 48.5 months (median 24 months). Data on the delay of BCC diagnosis in general are scarce. Husein-El Ahmed et al. found in a hospital setting an estimated mean delay of 19.8 months in the diagnosis of BCC. BCC located elsewhere other than the head and neck were significantly associated with a delayed diagnosis [27]. In our cohort, the initially misdiagnosed ulcerated BCCs were all misinterpreted as chronic venous leg ulcers. Chronic venous insufficiency has a high prevalence with an estimated 25% of the population showing clinical signs of CEAP (clinical–etiological–anatomical–pathophysiological) stage C2–C3 (varicose veins with or without edema) and 5% of the population showing trophic skin changes with or without ulcers (CEAP stage C4–6) [28]. Concomitant clinical signs of chronic venous insufficiency and positive duplex sonography may lead to a premature clinical misinterpretation of leg ulcers. This is underscored by a retrospective analysis of 60 patients with an initial diagnosis of venous ulcers due to clinical signs like varicosities, edema, and hyperpigmentation; 25% of these cases were finally malignant ulcers [29]. Atypical localization and discrepancy between the stage of chronic venous insufficiency and the occurrence of a chronic ulcer can help with differentiation [19]. In our cohort, two out of the four BCC falsely diagnosed as venous leg ulcers were located at the lateral gaiter area or lateral lower leg, which are atypical sites for venous leg ulcers [30]. Venous stasis as a predisposing factor for the development of BCC is controversially discussed [31,32,33].

The mean duration of chronic ulcers until the diagnosis of SCC in our study was 65 months, with a range of 3 to 240 months. Excluding the two cases with supposed malignant transformation, the mean diagnostic delay for SCC presenting as chronic ulcers was 36.7 months (median 24 months). This is longer compared with the data by Renzi et al., who found a mean delay in the diagnosis and treatment of SCC in all sites of 22.4 months with a mean patient delay to seek medical advice of 11.4 months [34]. Half of the SCCs in our study had a diameter greater than 2 cm. A delay in diagnosis and treatment increases the risk of SCCs greater than 2 cm in lesion diameter [35], resulting in an increased risk for metastasis and local recurrence [36,37].

A histological work-up of the tissue biopsy eventually proves the diagnosis of cutaneous malignancy. The proper time to make a biopsy in a chronic leg ulcer is controversially discussed. Recommendations vary regarding whether to biopsy all ulcers with a duration of more than 4–6 weeks to 4 months of non-healing wounds, despite adequate wound management [38,39,40,41]. Immediate histological evaluation to rule out malignancy should be performed in ulcers with exophytic growth, irregular wound edges, excess granulation tissue, or excessive bleeding [8,19,41,42].

There are some possible limitations in this study. The main limitation is the retrospective nature of this study. Data about first manifestation of ulcers depend on the information given by the patients or their relatives. This may lead to a bias of the time until correct diagnosis. Because of the retrospective character of the study, data about comorbidities may not be complete and interesting information about symptoms like pain or bleeding may be lacking. Therefore, we decided not to further analyse the data about symptoms like pain or bleeding as possible diagnostic clues. The fact that pain is not documented in the patients’ data chart does not mean that the patient had no pain. A further limitation is the referral bias in tertiary centres. The number of ulcerated tumours may be increased because patients in advanced tumour stages may be more often referred to a tertiary centre. Thus, the prevalence of malignant tumours imitating chronic leg or foot ulcers and the number of initially misdiagnosed cases in our study may be overestimated.

A prospective, multicentre study including chronic leg or foot ulcers existing for no longer than three years, in order to exclude secondary malignancies, as proposed by Baldursson B et al. [17], could help evaluate new criteria so as to uncover malignant tumours presenting as chronic leg or foot ulcers.

## 5. Conclusions

Malignant ulcers presenting as chronic ulcers are rare, but often lead to misdiagnoses. Therefore, repeated clinical re-evaluation of the diagnosis and biopsies in cases of non-healing wounds despite adequate wound management are crucial to detect ulcerated skin malignancies and to prevent the delay of the appropriate therapy. Chronic ulcers with atypical clinical aspects or atypical locations should arouse suspicion and a biopsy should be performed in a timely manner.

## Figures and Tables

**Table 1:** Case characteristics.

Case Characteristics			
	Total (*n* = 26)	Misdiagnosed (*n* = 12)	Correctly Diagnosed (*n* = 14)
Age, y			
Mean	78	78	77
Median	80	80	79
Range	57–97	57–97	59–88
SD	8.9	9	9.2
Sex			
Female	12	6	6
Male	14	6	8

SD—standard deviation.

**Table 2:** Misdiagnoses as chronic leg or foot ulcers of different origins (*n* = 12) and distributions according to tumour type.

	Posttraumatic	Venous	Arterial	Mixed	Unknown Aetiology
MM	2	-	1	-	1
SCC	1	1	-	1	1
BCC	-	4	-	-	-

MM—malignant melanoma; SCC—squamous cell carcinoma; BCC—basal cell carcinoma. - = no one.

## Data Availability

Not applicable.
